# “Everybody wants to coach it, but fewer know how to do it”: a qualitative study of stakeholders’ perspectives on mental skill development in youth sports

**DOI:** 10.3389/fspor.2025.1633943

**Published:** 2025-09-12

**Authors:** Philippe O. Müller, Ulrike Schwarzer, Dave Collins, Renato Frey, Jörg Spörri

**Affiliations:** ^1^ Sports Medical Research Group, Department of Orthopaedics, Balgrist University Hospital, University of Zurich, Zurich, Switzerland; ^2^ University Centre for Prevention and Sports Medicine, Balgrist University Hospital, University of Zurich, Zurich, Switzerland; ^3^ Cognitive and Behavioral Decision Research, Department of Psychology, University of Zurich, Zurich, Switzerland; ^4^ Grey Matters Performance Ltd., London, United Kingdom; ^5^ Human Performance Science Research Group, Institute for Sport, Physical Education and Health Sciences, The University of Edinburgh, Edinburgh, United Kingdom

**Keywords:** athletes, athletic performance, psychological resilience, mental health, human development

## Abstract

**Background/objectives:**

In recent years, the role of psychological training in youth sports has received increasing attention in both research and practice. The development of mental skills is important for sporting excellence, as it improves performance and helps athletes grow in their personality. This study aimed to explore the views of a wide range of stakeholders in youth sports on current practices, challenges, and opportunities related to mental skill development programs. With its comprehensive, multi-perspective approach, it provides novel insights into how such programs are perceived and implemented across different roles in youth sports.

**Methods:**

Fifteen semi-structured qualitative interviews with coaches and sport psychologists from multiple countries were conducted. The data were transcribed verbatim and the primary inductive process was based on constant comparative analysis employing the principles of Grounded Theory. In addition, a subsequent deductive process involved qualitative content analysis. Finally, the inductively and deductively derived outcomes were compared and the key conclusions of the study were formed via abductive reasoning.

**Results:**

The interviewees highlighted the importance of organizational structure, personal interactions and standardized processes for the successful implementation of mental skills development programs. They considered clearly defined roles and responsibilities to be crucial to the systematic development of mental skills in athletes. The most common barriers identified were limited financial and human resources. In addition, the implementation process was described as often hampered by a lack of knowledge among decision makers about the benefits of systematically developing mental skills. Improved interdisciplinary collaboration (e.g., between teachers, coaches, parents and sports psychologists) was reported to be key to optimizing resource allocation and facilitating the development of mental skills.

**Conclusion:**

The results suggest that systematically integrating mental skills development into youth sports requires institutional support, stakeholder engagement, and interdisciplinary collaboration to ensure its application, implementation, and sustainability. Accordingly, mental skills development programs should be embedded in organizational strategies and policies. This clarifies responsibilities and allows for the development of appropriate measures. A key aspect of such integration is providing knowledge about successful, healthy mental skill development pathways and appropriate intervention measures to all stakeholders.

## 1 Introduction

Mental factors play a crucial role in the development of superior sports performance (1–3). However, the development of mental skills is important not only for optimizing performance and achieving sporting excellence but also in the development of the athlete's personality and protection of physical and mental health (4, 5). In addition to the challenges encountered in everyday life, competitive athletes must address the challenge of balancing sport with other areas of their lives (6, 7). They also have to learn to cope with sport-specific stressors such as injuries, pressure situations, career transitions, motivational fluctuations, and competition at a young age (8).

Consequently, the psychosocial system of athletes is a complex structure consisting of several interacting and influential environmental factors and structures that challenge and impact their long-term development. It is therefore important to take a holistic and ecological view (7, 9). Accordingly, the development of mental skills should be congruent with both the sporting and nonsporting environments and embedded within the athlete's basic social structure (10, 11). This latter goal encompasses the immediate social grouping (coaches, parents and peers) as well as the wider sports environment (clubs, associations) (12).

Existing research mainly refers to developmental and sport psychology models and derives frameworks and recommendations for the design of mental training programs (13–15). However, effective mental skill development should also be related to a person's personality, activity, gender and age (11) and hence should be as individualized and sport specific as possible (16). In addition, a focus on the long-term, continuous, systematic and holistic development of young athletes has been suggested to ultimately be more successful than a performance-oriented approach with early specialization (10, 17). Therefore, in addition to mental training aimed at achieving optimal performance, personality development and coping strategies should also be part of mental skills development programs (11).

These additional aspects, which are not primarily performance oriented, can be found in existing mental skills development programs, such as “*The First Tee”* (18) in golf, “*5C's”* (19) in football, and “*UNIFORM”* (20) in high school sports. In a similar context, previous research has highlighted that the initial focus should also be on the development of fundamental skills such as motivation, self-awareness, willingness to perform, and self-confidence (13). These skills help young athletes learn and use different strategies, such as relaxation techniques, imagery, and concentration exercises (21). Accordingly, the long-term, continuous, systematic and holistic development of mental skills should build resources that can both apply to and be transferred from the sporting context (22). Even if athletes do not remain in competitive sports, they can still benefit from the skills they have learned. Systematic mental training in youth sports is therefore at least as important as physical training.

Despite widespread advocacy for mental training programs (23, 24), however, research on their practical implementation remains limited. Several critical gaps persist, including uncertainties regarding the structure and delivery of mental training in youth sports, the identification of age-appropriate content, the designation of responsibility for providing mental training, and the necessary professional training for those involved. Furthermore, the challenges of integrating mental skills training into existing sports programs have not been fully addressed. While some studies have examined the perspectives of athletes and other stakeholders on mental skills in general (25, 26), on life skills transfer to sport (27–29), and on sport psychology consultancy (30–32), there is a significant shortcoming regarding athlete and stakeholder perspectives on the long-term development of mental skills (33). Understanding their experiences and insights is critical to the development of effective, sustainable, and evidence-based mental training programs that are tailored to the realities of youth sports. In this context, the Performance–Outcome–Process (POP) model offers a helpful framework for structuring mental skills development (34). By distinguishing between long-term performance goals, intermediate psychological outcomes, and underlying developmental processes, the POP model promotes a comprehensive and practically relevant approach. This structure supports the integration of mental skills into athlete development by emphasizing the need to proactively teach and refine foundational psychological processes that can sustain progress across a young athlete's journey.

To address these remaining gaps in the literature, this study explores youth sports stakeholders' perspectives on the implementation of mental skills development programs in youth sports. More specifically, it examines current practices, challenges, and opportunities to systematically enhance mental skills from a long-term perspective. By integrating stakeholder insights, our study may bridge the gap between theoretical recommendations and real-world application, thereby contributing to a more structured and evidence-based approach to mental skills development in youth sports.

## 2 Methods

### 2.1 Research paradigm and study design

**Figure 1:**
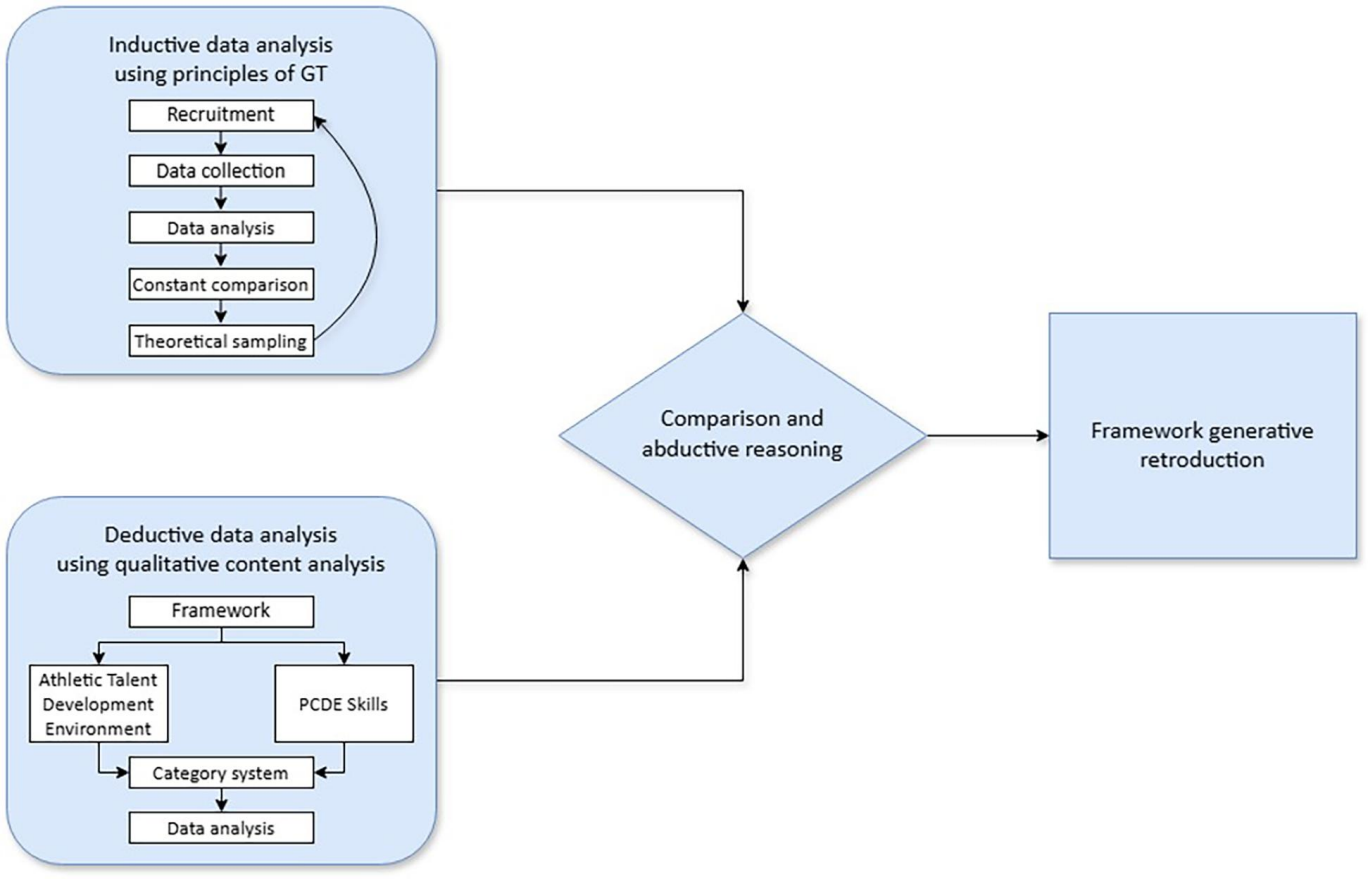
Visualization of the multistage research paradigm and study design.

To address the holistic nature of our research interests adequately, a multistage research design was selected, as illustrated in Figure 1. The study followed the principles of interpretative social research and emphasized the subjective experiences and opinions of the stakeholders interviewed.

In the first research stage, an inductive analysis of the data facilitated the construction of new concepts and theories (35). The methodological approach was based on the principles of Grounded Theory (GT), incorporating iterative data collection and analysis, constant comparison and theoretical sampling (36–38). Specifically, the systematic analysis process involved the interpretation and correlation of experts' opinions, meanings and practical findings (37). The analysis prioritized the close examination of the source material through various coding steps rather than relying solely on the researchers' prior knowledge. In addition, the underlying concepts of practical knowledge and its interpretation were based on a constructivist approach (39, 40). The reporting structure follows the Consolidated Criteria for Reporting Qualitative Research (see Supplementary File 1) (41).

Given the existence of established theories in the field of talent development, in the second research stage data were analyzed deductively via qualitative content analysis (42). The participants' responses were categorized into predefined themes on the basis of two frameworks: the *Athletic Talent Development Environment (ATDE)* (7) and the *Psychological Characteristics of Developing Excellence (PCDE)* (1). In the final research stage, abductive reasoning was carried out by comparing the inductive and deductive results.

### 2.2 Participants and recruitment

The interviewees for this research were stakeholders involved in the implementation of mental skills development programs, such as coaches, sports or performance psychologists, (sports) educators, teachers, or researchers. These stakeholders are characterized by special technical and processual knowledge and integrated experience in this specific field (43).

**Figure 2:**
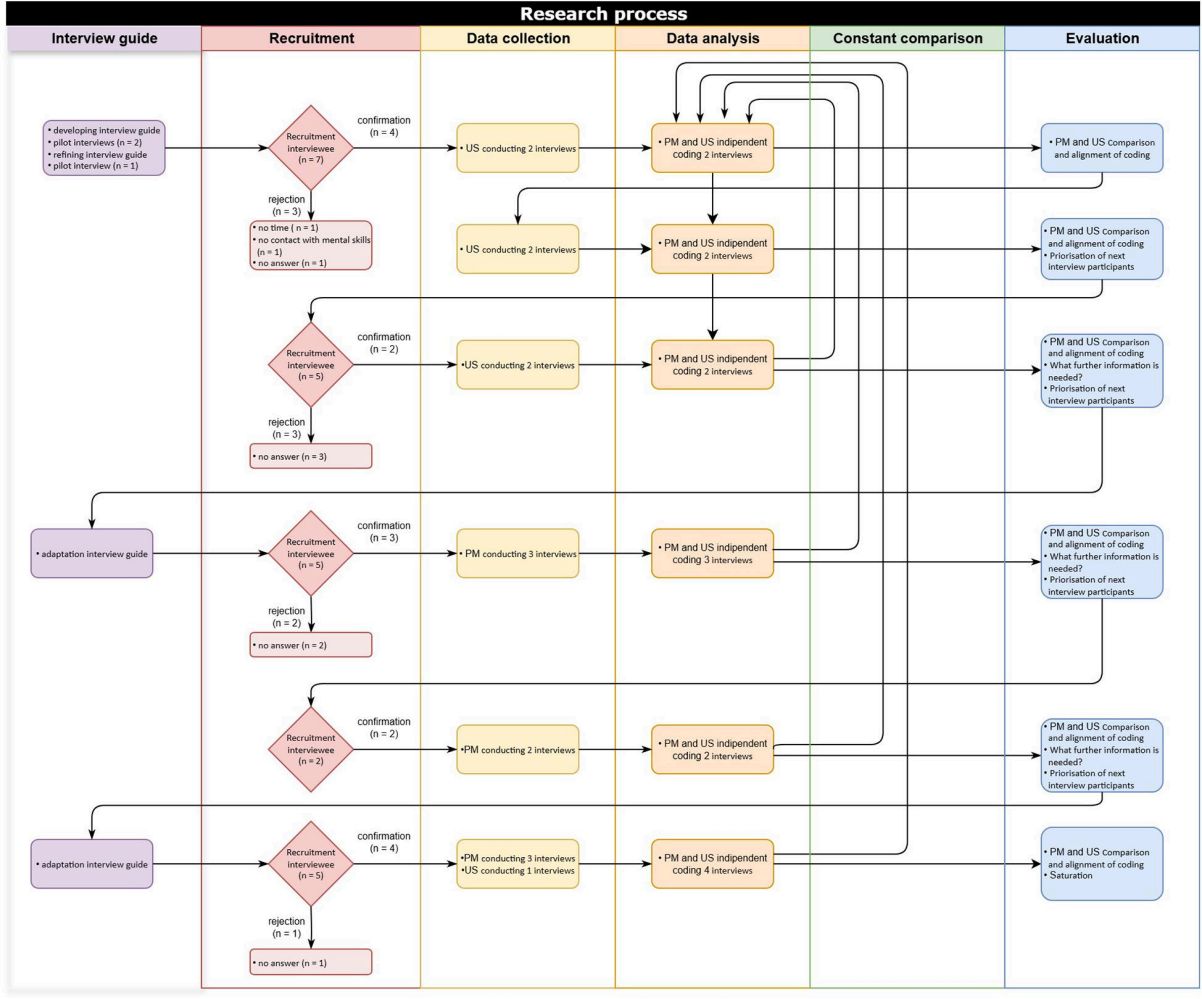
Flow diagram showing the research process for inductive data analysis**.**

Figure 2 illustrates the exact recruitment process in more detail. Recruitment was conducted in two ways: (1) The authors' own network of contacts provided information about potential interviewees with whom the authors were not connected. (2) A respondent-driven sampling approach was chosen for the subsequent recruitment process; this means that at the end of each interview, interview participants were to suggest further interviewees. The selection and final inclusion of any interviewees were discussed and agreed by the research team. Our aim was to maximize diversity in terms of profession, experience, nationality, sports, sport setting (individual and team), institution (school, academy, association, club), and gender.

In the initial recruitment step, 7 participants were invited, and 4 agreed. At the end of the interview, these participants were asked to suggest additional stakeholders to be interviewed, along with their contact details. Using this respondent-driven sampling approach, further participants were invited. In the second recruitment step, 2 out of 5 invited participants agreed to be interviewed; in the third recruitment step, 3 out of 5 agreed; in the fourth recruitment step, 2 out of 2 agreed; and in the fifth recruitment step, 4 out of 5 agreed. This resulted in a total of 15 interviews. The participants were contacted via email, and provided with details in the form of a study information sheet. There were no prior personal relationships between the participant and the researchers who conducted the interviews. The present study protocol was assessed by the Cantonal Ethics Committee KEK Zurich (BASEC No. Req. 2023-001330) and was judged not to fall within the scope of the Human Research Act (HRA). This meant that authorization from the ethics committee was not needed and that written informed patient consent was waived.

### 2.3 Data collection

Data were collected between February and September 2024 through individual semi-structured interviews in accordance with the recommendations of Gläser and Laudel (44). The interviews were conducted online by the researchers PM (8 interviews) and US (7 interviews), in English, German, or Dutch via Microsoft Teams. The research team designed an interview guide to ensure comparability of the interviews (45). To ensure the linguistic equivalence of the interview guide across different languages, a back-translation was performed.

The interview guide was divided into three sections: (1) In the *introduction section*, the stakeholders introduced themselves and described their area of responsibility and their relationship with mental skills training in youth sports. This concrete introduction was important for focusing on the main themes of this study and placing the interviewees at the center as experts. (2) In the *main section*, the participants explained their experiences with various topics in much more detail and independently. This allowed the interviewer to understand the different perspectives and gain deeper insight into the participants' perceptions. Issues raised could then be explored in more depth by asking specific questions (44, 46). (3) The *conclusion section* contained questions that gave respondents the opportunity to summarize what they felt were the most important elements (44, 46). This led to a reflective process in which they were able to reevaluate, reinforce or relate what they had mentioned in the main section.

Two pilot interviews with a mental coach and a sports psychologist from the personal network of the researcher US were conducted to test the process, technical details, and interview guide in advance (45). The interviewees' feedback and recorded interviews were discussed between US and PM regarding the process, completeness and conduct of the interviews, resulting in one question in the main section being moved to the conclusion section and one question in the introduction being made more general. Data from the pilot interviews were not included in the data analysis.

Based on the concepts of iterative data collection and theoretical sampling, the guideline was adapted after each round of data collection and analysis during the evaluation phase. For example, during the first round of further development, direct questions about gender differences and responsibility for mental skills training were added. The second iteration included specific questions about the necessary steps for successfully implementing mental skills programs.

After 15 interviews, no new categories emerged during data collection and no new codes were generated, indicating data saturation, and theoretical saturation was also reached (47).

### 2.4 Data analysis

**Figure 3:**
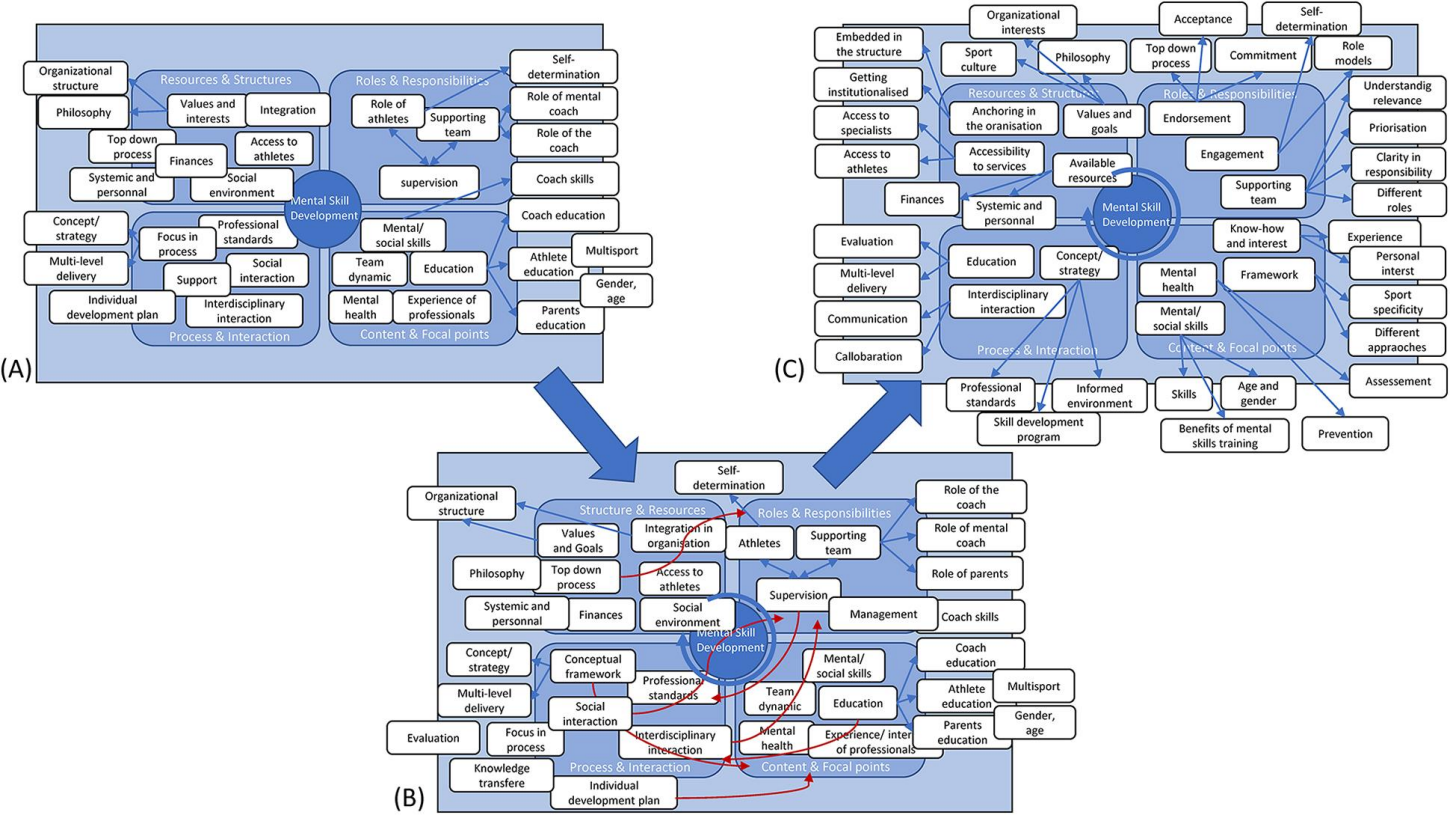
Visualization of the process of constant comparison and concept development. **(A)** Status after 6 interviews. Concept development by forming categories and relationships. **(B)** Status after 9 interviews. Further development of concepts and categories. **(C)** Status after 12 interviews. Clarification and refinement of concepts and categories.

The inductive data analysis process is illustrated in Figure 2. The interviews were recorded and transcribed verbatim. The transcripts were checked for accuracy against audio recordings but were not reviewed by the interviewee at this stage. The coding process aimed to identify both similarities in the data and higher-level concepts (48). The data were open coded (26) by coding software (MAXQDA V.24.2.0). In the first phase, the authors familiarized themselves with the content by reviewing the transcripts and highlighting areas of interest (36, 37). Two researchers (US and PM) independently coded the first two transcripts, and the resulting codes were compared and discussed. After alignment, the next two interviews were coded independently and discussed to establish a consistent approach to the categorization system, resulting in a hierarchical structure of codes and categories (26). After the next two coded interviews, a third researcher (JS) who was not familiar with the data, was consulted to discuss the comprehensibility of the preliminary results. The comparison of the coding between US and PM took place after an additional 3 interviews and then 2 and the last 3 interviews. The final results were again discussed with JS. In accordance with the constant comparison approach, new data were collected or analyzed to complete, specify and verify the categories identified. This ongoing comparison of the existing material aimed to avoid premature assumptions, unspecific, banal or redundant categories, uncover possible data gaps, answer the research question and sharpen the development of ideas in terms of theory formation (37, 38). When the data were reanalyzed, no inconsistencies or contradictions with the categories and concepts were found. Finally, to identify the main concepts, categories and subcategories and to create the final conceptual model of the analysis, the results were structured considering similarities, differences and connections. The process of constant comparison with the development of the concepts is shown in Figure 3.

### 2.5 Trustworthiness of the data

In qualitative research, ensuring trustworthiness is critical to the scientific integrity and credibility of the research findings, and the results authentically reflect the experiences and perspectives of the participants and are methodologically sound (49). The trustworthiness of the results of this study was ensured through several measures. First, the data were collected through semi-structured interviews, which were analyzed via both inductive and deductive methods (50). As a constructivist grounded theory approach was adopted in this study, the research team was aware that the data collected were a product of both the researcher and the subject. To minimize bias and ensure sound interpretation, the data were analyzed independently by two researchers. The results were then cross-checked to ensure consistency and rigor. In addition, the results were critically discussed with a third independent researcher who was not familiar with the raw data. This person acted as a critical friend, asking specific questions and challenging interpretations to uncover blind spots or potential biases. Furthermore, the results (i.e., the inductively derived main concepts and categories with brief descriptions) were then sent to the participants for reflection, who were asked to provide feedback on four questions aimed at the accuracy, completeness and appropriateness of the interpretation (51). This gave them the opportunity to reflect on their own experiences and make additional comments or considerations, which were then incorporated into the overall results. Importantly, the final judgment of transferability rests with the readers, who should assess it on the basis of the contextual information provided (52). This highlights the necessity for a critical and reflective transfer of the results to other settings.

## 3 Results

A total of 15 interviews were conducted, with coaches (*n* = 3), sports psychologists (*n* = 5), (sports) educators (*n* = 3), researchers (*n* = 2), and teachers (*n* = 2), eight of whom were women. The interview participants were from a wide variety of nationalities: Canada (*n* = 1), New Zealand (*n* = 1), Switzerland (*n* = 1), Austria (*n* = 1), the Netherlands (*n* = 1), Germany (*n* = 1), Great Britain (*n* = 3), the USA (*n* = 2), Iceland (*n* = 1), and Finland (*n* = 3). The mean years of experience was 14.1 years. One participant worked with young athletes between the ages of 8 and 12. The other participants all worked with athletes between the ages of 13 and 20. They worked in public schools (*n* = 2), sports academies (*n* = 5), clubs (*n* = 4), national Olympic training centres (*n* = 2), and national sports associations (*n* = 2). The clubs (*n* = 4) were single-sport organizations, whereas the other organizations (*n* = 11) were multisport organizations (winter sports = 4; summer sports = 7). To ensure the anonymity of our participants, no further details are provided. The average interview length was 49 min (range: 34–70 min).

### 3.1 Results identified through an inductive process using principles of GT

**Figure 4:**
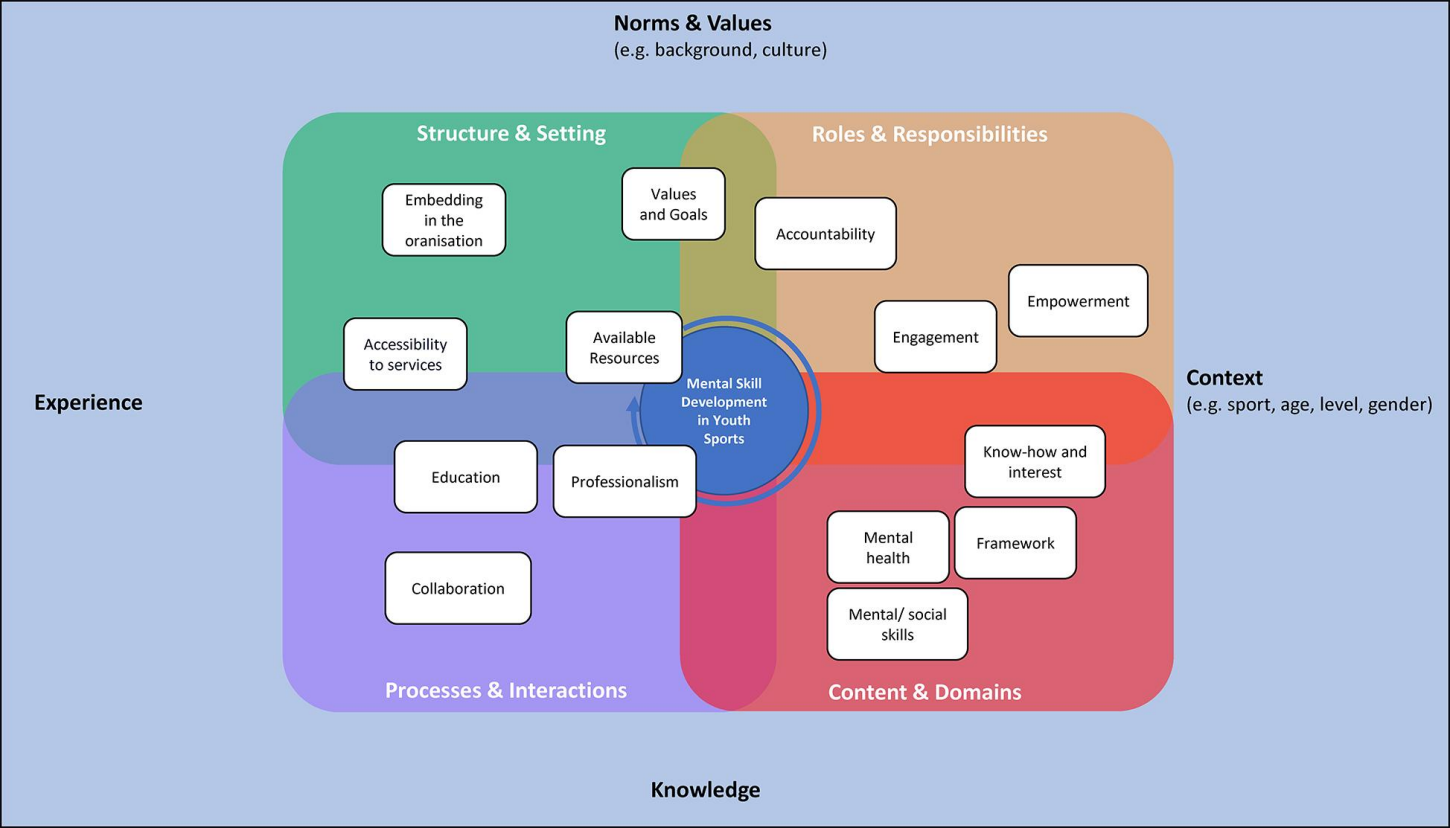
A multifactor framework for mental skill development in youth sports. The colors group the categories and subcategories into the four main concepts. Starting from the top left of the figure and moving clockwise, green refers to structure and setting, orange refers to roles and responsibilities, red refers to content and domains, and purple refers to processes and interactions.

As shown in Figure 4, four main concepts emerged from the consolidation of the expert interviews, which represent the conceptual levels of their current practice in the implementation of mental skills training programs in youth sports. There are multiple relationships between the core concepts and categories, although clear boundaries are apparent.

#### 3.1.1 Structure and setting

This main concept addresses the organizational and structural framework conditions, as well as the fundamental interests and values of sports organizations that can promote or hinder the development of mental skills in youth sports. The concept comprises the following four categories: values and goals, anchoring in the organization, accessibility to services and available resources. Table 1 contains representative quotes to illustrate the categories related to the first concept, 'structure & setting'.

**Table 1:** Quotes to illustrate the categories related to the first concept, ’structure & setting’.

Category	Illustrative quotes
3.1.1.1 Values & Goal	“First of all, the stuff, including all the physiotherapists and special coaches, let's say goalie coaches, and strength and conditioning coaches, and all of the stuff, they share the same idea of human being. We have the same philosophy.” participant 14 “So the barriers is that the leadership maybe don't understand it enough.” participant 7 “Each organization has a responsibility to develop their long term plans.” participant 1
3.1.1.2 Available resources	“I have mentioned it already. Is the time, time barrier, which ultimately comes down to a financial barrier. If I can get more time to work with coaches and teachers, as well as time to develop content and programs, then I can do it.” participant 11 “It has to be developed and that costs money and money is not there.” participant 5 “There's a business challenge. For example, when I want a new mental coach, I have to build a business case.” participant 10
3.1.1.3 Embedding in the organization	“So, we have a hierarchy within the department. We have a structure based on experience and expertise.” participant 10 “The vast majority of schools, I'd say like 99% plus of schools don't have that programming. So that would be a great start just getting one person at each school. That could be a resource, but if it's a long way for them” participant 11 “The work we are doing there is integrated to school life and it's quite small what we are doing. But it's one part of the work.” participant 13
3.1.1.4 Accessibility to services	“So access to athletes is a problem.” participant 2 “It's very difficult to get athletes of the of different spots on the same schedule, because the training programs are very different.” participant 10 “We don't have something that every athlete can use. It's more like, if you are in a young national team, then you are able to work with a mental trainer.” participant 15

##### 3.1.1.1 Values and goals

Stakeholders highlighted the importance of sport organizations developing a common philosophy to integrate mental training into their sport culture and putting the athlete at the center. This requires the definition of values and goals.

##### 3.1.1.2 Available resources

Allocating sufficient time, personnel and financial resources for implementing mental skill development was key for the stakeholders. Time and effort are crucial factors not only for the daily training of (youth) athletes but also for the development of structured mental training concepts at an organizational level. However, financial resources were described as a key issue in all countries and settings, both in terms of funding priorities within the organizations and the overall financial situation in sport.

##### 3.1.1.3 Embedding in the organization

To facilitate sustainable mental skills development, stakeholders considered it essential that mental training programs be institutionalized and embedded within the organizational structure, thus ensuring their integration into the overarching strategy.

##### 3.1.1.4 Accessibility to services

Direct access to mental services was named a central approach to be integrated into the organizational structures of sports federations. This included easy access for athletes to specialists, as well as the structural conditions for training programs to reach athletes.

#### 3.1.2 Roles and responsibilities

The complex setting of roles and responsibilities, with a major focus on the contributions and challenges faced by different groups (coaches, athletes, support teams, and organizations), was one main concept inductively derived from the stakeholder interviews. Stakeholders highlighted the importance of a cohesive, transparent, and supportive framework to ensure athlete well-being and success. Table 2 contains representative quotes to illustrate the categories related to the second concept, “roles & responsibility”.

**Table 2:** Quotes to illustrate the categories related to the second concept, “roles & responsibilities”.

Category	Illustrative quotes
3.1.2.1 Accountability	“Yeah, the institutional commitment is significant.” participant 11 “That's also very important for us to get to know what do we need for an environment and also the commitment from the system.” participant 1 “It's been really welcoming, and the directors are really driving it. They talk to the coaches about how psychology can support them and their athletes.” participant 2
3.1.2.2 Engagement	“There's some coaches and some directors in some sports don't see the value, you know, make it a little more difficult to schedule” participant 10 “So the barriers is that the leadership maybe don't understand it enough. So when the leadership don't understand it or don't value it then, the coaches, like myself, we choose, maybe not to do it as much as we could do because we also want to keep our job and conform to our environment.” participant 7 “So we will plan and try and get clarity on which staff members are responsible and the specialist will be responsible for those areas of development.” participant 8
3.1.2.3 Empowerment	“You're just here to support me and I'll tell you what I need.” participant 2 “So we have to rethink and look at how we can bring more self-determination into training. At all levels, too. And it's not the athlete who comes to practice and the coach says what we're going to do, but that we somehow take it seriously. We used to call this autonomy. The responsible athlete.” participant 6 “I'm trying to get the coaches more involved as well and the coaches are really willing” participant 2

##### 3.1.2.1 Accountability

Specifically, top-down processes, acceptance and commitment were mentioned as crucial. In this context, accountability emphasizes responsibility and ensures that leadership decisions are implemented, that acceptance is translated into active engagement, and that commitment is consistently maintained.

##### 3.1.2.2 Engagement

Engagement was described as crucial for prioritizing tasks, understanding the relevance of mental skill demands, and clarifying roles and responsibilities. This emphasizes the need for active participation and ownership in the process of implementing mental skill development in youth sports.

##### 3.1.2.3 Empowerment

Empowerment was considered another central aspect with respect to mental skill development, as it emphasizes the autonomy of (youth) athletes and promotes a growth environment through the proactive involvement of coaches, support teams and parents. Coaches and parents should be encouraged to be open to the development of mental skills and should provide support, whereas athletes should be empowered to take control of their own mental skill development. This collaborative approach can create a holistic, athlete-centered culture where everyone is committed to the development of mental skills and mental health.

#### 3.1.3 Content and domains

With respect to the conceptualization and content of mental training programs, know-how and interest, framework, mental health, and mental and social skills were key categories mentioned by the stakeholders. Table 3 contains representative quotes to illustrate the categories related to the third concept, “content & domains”.

**Table 3:** Quotes to illustrate the categories related to the third concept, “content & domains”.

Category	Illustrative quotes
3.1.3.1 Know-how and interest	“It's all very *ad hoc*. It's just like that. It depends on if the coach is interested, then they might do some stuff on it, and if the coach isn't then they probably won't.” participant 7 “I believe that coaches who are trained and who have been working in the field for a long time develop a certain pedagogical skill, some more, some less. And with experience you can perhaps handle certain things better.” participant 6 “I am certainly also sensitized. For this reason, I want to create a good and healthy atmosphere so that the children can develop in a healthy way, even when there are challenges.” participant 3
3.1.3.2 Framework	“In terms of theoretical orientation, I would say I'm a bit eclectic. I come from a coaching background and so coaches take what works and discard what doesn't. So, but on the whole, I would call it a blend between cognitive behavioral theory and mindfulness. I find those are the two areas I draw in the most in developing that curriculum.” participant 11 “We don't have a framework for a reference. We talk about character and the importance of our of our players having it, which is essentially an ability to learn. We want them to be good learners.” participant 7 “I think then also you have to choose a framework to work with or develop your own if you're not happy with those frameworks who are available.” participant 12
3.1.3.3 Mental health	“But I think that, in this time, the information we're providing is more about how to maintain or create good mental health.” participant 13 “They will also provide support and guidance for the young players, both on the field and off. They will work with the player care team to promote player well-being and mental health.” participant 8 “I think sports are like a school of life, with a focus on high performance. If we can make them healthy and development-friendly, then we can apply these approaches to schools later on.” participant 3
3.1.3.4 Mental and social skills	“They are human beings, and you need to teach them useful skills that will benefit them for the rest of their lives.” participant 5 “We also always emphasize that everything we do is relevant not just to sports, but to life in general.” participant 9 “In short, the value of this strategy is that it adds to people's toolbox of skills to help them deal with situations they face in sports and in life.” participant 12

##### 3.1.3.1 Know-how and interest

According to the stakeholders, the relevant theoretical and practical content of mental training is often determined by the expertise of the professionals responsible for designing the program. The personal experiences and interests of these professionals therefore strongly influenced the development of the programs.

##### 3.1.3.2 Framework

The concepts underlying mental training programs are very diverse, as the stakeholders indicated. Typically, several psychological models are combined and adapted to the specific conditions of the organization (structure, type of sport, etc.).

##### 3.1.3.3 Mental health

Numerous programs are currently implemented to maintain and promote the mental health of athletes. Mental health was frequently described as the foundation for optimal athletic performance by stakeholders.

##### 3.1.3.4 Mental and social skills

The mental and social skills emphasized in current development programs are determined by the underlying framework. According to the stakeholders, these skills are tailored to consider both age-related development and gender-specific differences. Furthermore, the positive effects of mental skills training in general were emphasized by some stakeholders. As such, training concepts are typically designed to enable young athletes to familiarize themselves with various mental tools in sports and make them usable for later life.

#### 3.1.4 Processes and interactions

In the context of the processes and interactions required for the successful implementation of mental skills development programs in youth sports, stakeholders have emphasized the importance of professionalism, education and collaboration. Table 4 contains representative quotes to illustrate the categories related to the fourth concept, “process & interaction”.

**Table 4:** Quotes to illustrate the categories related to the fourth concept, “processes & interactions”.

Category	Illustrative quotes
3.1.4.1 Professionalism	"So, to me, one of the things is support. Support should be done well and done right.” participant 1 “As I said, I think there's also an issue with who gets to work in this field. As I said, there have been a lot of charlatans occupying this sphere, which confuses the concept of quality.” participant 12 “And they have a performance development plan. So this is a plan for their own development. […] I think every three weeks, so four times the semester, they are also working with other people within the department to get feedback on a consistent basis.” participant 10
3.1.4.2 Education	“And then there would be education of the teachers. So that teachers could both, teach and guide students in building their self-awareness and developing the practices, the mental skill practices in multiple contexts.” participant 11 “So, we will provide internal coaching education to help coaches develop mental skills and other areas. This will develop the coaches’ awareness and coaching skills, teaching them how to deliver and support strategies collectively on the pitch to a team and how to provide effective feedback on an individual level.” participant 8 “I want mental health considerations to be integrated into the practice environment wherever possible. There's time for the classroom, but I want it in practice. I want it through the coaches, and I want it through physical coaching. I want it through sports coaching. That's ideal for me.” participant 10
3.1.4.3 Collaboration	“If I value mental skills and understand them, I don't have a lot of power or control to commit them with the players. If all of my colleagues don't understand them or don't value them, we can't do it because I'm just one person.” participant 7 “And of course, athletes sometimes run into issues and then they also go to a psychologist. But that's much more integrated into the program. The psychologist and the coach also talk to each other and adapt the program to the needs of the athletes.” participant 5 “The strength and conditioning coach, athletic coach, physiotherapist, and mental performance coach should have regular opportunities to meet. This will ensure that all adults working directly with individual athletes have a comprehensive and consistent approach” participant 11

##### 3.1.4.1 Professionalism

A clearly defined strategy was, according to the stakeholders, essential for success. It was emphasized that professional standards need to be constantly developed and improved to meet changing requirements. Consequently, the whole environment (coaches, athletes, teachers, parents, etc.) is also informed and pulled in the same direction. This is the key to effective collaboration and helping athletes optimize and apply their mental skills in an increasingly professional manner.

##### 3.1.4.2 Education

To best support athletes, mental skills development programs should, according to stakeholders, be promoted in different ways, such as coach education, athlete education, and parent education. These education programs should be implemented at different levels and by different delivery formats, such as seminars, practical workshops or individual coaching. The evaluation of these education programs is essential for the continuous improvement of content and methods.

##### 3.1.4.3 Collaboration

Stakeholders highlighted that the implementation of mental skills development programs requires multidisciplinary and interdisciplinary cooperation. Direct communication is essential for sharing relevant information and maximizing synergies. Collaboration is necessary to identify and address specific needs and to optimize the use of available resources.

### 3.2 Results identified through a deductive process using content analysis

Following the inductive process, a deductive content analysis was used to categorize the stakeholders' responses into predefined themes on the basis of two common frameworks: the Athletic Talent Development Environment (ATDE) (7) and the Psychological Characteristics of Developing Excellence (PCDE) (1).

#### 3.2.1 Athletic talent development environment (ATDE)

The athletic talent development environment (ATDE) is a critical system that influences athletic performance and personal development. It is influenced by structural, interpersonal, and cultural factors, as highlighted in the holistic ecological framework (53). Through the analysis, the stakeholders consistently highlighted the relevance of structural and organizational aspects of their environments, emphasizing the importance of resources, support, and cultural integration. As Table 5 shows, the analysis identified six key clusters for ATDEs, representing structural and interpersonal factors that create a conducive environment for athlete development.

**Table 5:** Themes, clusters, and categories related to the ‘athletic talent development environment (ATDE)’.

Theme	Cluster	Categories
3.2.1.1 Athlete resources	Preconditions - time, personal, and financial resources	Funding
3.2.1.2 Organizational support	Preconditions - structural Resources	• Structured programs • Institutional policies • Embedded within the organizational structure • Access to providers
3.2.1.3 Coach-athlete dynamics	Microenvironment - Athlete Domain	• Coach-athlete interaction • Mentorship • Coaching culture
3.2.1.4 Team and peer dynamics	Microenvironment - Athlete Domain	• Peer group identification • Team bonding • Parents support • Emotional-social dynamics • Support team
3.2.1.5 Transition support	Outcomes - transition success	• Individual development plan • Dual-career support • Personal growth
3.2.1.6 Cultural integration	Contextual framework - cultural influences	• Cultural values • National sport system • Sport-specific culture

##### 3.2.1.1 Athlete resources

With respect to athlete resources, three clusters emerged in the stakeholder interviews: “time resources”, “personal resources” and ‘financial resources. The stakeholders particularly emphasized that a lack of time was a major challenge in the implementation of mental skills development programs, as balancing training and education schedules can often be an obstacle. Personal resources were also mentioned as a potential implementation barrier, with the greatest challenge being the availability of mental coaches/sport psychologists in relation to the number of athletes. In addition, financial resources were described as playing a crucial role in implementing mental skills development programs, as insufficient funding limited access to mental training and its providers. Taken together, these findings highlight the importance of available resources for an athlete's long-term mental skill development.

##### 3.2.1.2 Organizational support

The theme of organizational support is represented by the cluster of “preconditions–structural resources”. Stakeholders emphasized that structured programs not only provide clear developmental pathways but also create a stable environment, ensuring predictability in an athlete's journey. The role of institutional policies and organizational frameworks was described as fundamental to maintaining consistency in athlete development. Additionally, systematic organizational access to service providers and institutional backing were considered essential in equipping athletes with professional support and necessary resources, ultimately facilitating sustained development and career longevity.

##### 3.2.1.3 Coach-athlete dynamics

The theme of coach-athlete dynamics is reflected in the cluster of “microenvironment–athletic domain”. Stakeholders frequently referred to coach-athlete interactions as a critical factor influencing both performance and mental health. The presence of mentors was particularly noted, as coaches were considered not only as technical experts but also as key figures providing guidance and emotional support. In addition, the overall coaching culture played an important role in shaping the mental training environment, strengthening mental health and encouraging long-term commitment to sport. These findings highlight the key influence of coaches on an athlete's personal development, performance and health.

##### 3.2.1.4 Team and peer dynamics

The theme of team and peer dynamics is covered by the cluster of “microenvironment–athletic domain”. The stakeholders emphasized that identifying with a peer group fostered a sense of belonging, which in turn reinforced motivation and engagement. The importance of team bonding was frequently mentioned, as strong interpersonal relationships contributed to psychological resilience. Additionally, parental support and the presence of a support team play crucial roles in maintaining emotional well-being. Overall, the emotional-social dynamics within an athlete's environment were seen as instrumental in sustaining motivation and facilitating skill development.

##### 3.2.1.5 Transition support

The theme of transition support is situated within the cluster of “outcomes–transition success”. The stakeholders identified the transition from youth/junior to elite/senior levels as a particularly challenging phase, requiring structured guidance in terms of mental skills. The implementation of individual mental skills development plans was seen as crucial in helping athletes navigate this shift effectively. Additionally, dual-career support was mentioned to play a vital role in balancing sports commitments with academic or professional pursuits. Beyond performance, the youth/junior to elite/senior transition is considered to foster personal growth, equipping athletes with mental and social skills essential for both their sporting careers and life beyond competition.

##### 3.2.1.6 Cultural integration

Cultural integration is reflected by the cluster of “contextual framework–cultural influences” that emerged in the interviews. The stakeholders emphasized that cultural values significantly influenced their experiences, and shaped their attitudes, behaviors, and team dynamics. It was also stressed that a well-aligned national sport system strengthens cohesion and provides a structured foundation for development, whereas discrepancies between cultural expectations and training environments may lead to challenges. Additionally, the influence of sport-specific culture plays a pivotal role in fostering a sense of identity and belonging, which ultimately influences long-term development and engagement in sport.

#### 3.2.2 Psychological characteristics of developing excellence (PCDEs)

The psychological characteristics of developing excellence (PCDEs) represent the psychological and behavioral attributes essential for sustained performance and well-being. This framework emphasizes mental skills as central to sporting and personal growth and enhances athletes' capacity to navigate the demands of their sports (10). Moreover, the interviewed stakeholders frequently named psychological skills critical enablers of success, highlighting attributes such as goal setting, resilience, and focus. Overall, seven clusters (Table 6) with relevant subcategories emerged from the analysis.

**Table 6:** Themes, clusters, and categories related to the “psychological characteristics of developing excellence (PCDEs)”.

Theme	Cluster	Categories
3.2.2.1 Psycho-behavioral skills	Mental skills	• Goal setting • Mental imagery • Self-talk • Relaxation • Self-regulation • Commitment • Motivation
3.2.2.2 Coping and resilience	Stress management	• Emotional regulation • Performance under pressure
	Adaptability	• Shifting strategies • Flexibility during setbacks
3.2.2.3 Motivation and engagement	Intrinsic motivation	• Self-determination • Task-based motivation
	Extrinsic motivation	• Coach-driven goals • Parental encouragement
3.2.2.4 Self-management	Time management	• Balancing dual careers • Training schedules • Daily planning
	Discipline	• Regular practice • Following a structured daily schedule
3.2.2.5 Social and emotional intelligence	Team collaboration	• Peer learning • Effective communication in teams
	Empathy	• Recognizing teammates’ struggles
3.2.2.6 Performance preparation	Imagery	• Visualizing winning routines • Practicing scenarios mentally
	Focus and attention	• Blocking distractions • Intense focus during routines
3.2.2.7 Personal growth	Resilience	• Overcoming injury setbacks • Emotional recovery from competition failures
	Self-confidence	• Positive affirmations • Confidence after achieving small goals

##### 3.2.2.1 Psycho-behavioral skills

The theme of psycho-behavioral skills is represented by one cluster that emerged in the interviews: “mental skills”. Within the mental skills category, the stakeholders emphasized the importance of goal setting, mental imagery, self-talk, relaxation, self-regulation, commitment, and motivation as foundational tools for sustaining high-level performance and fostering consistency in competitive environments.

##### 3.2.2.2 Coping and resilience

The theme of coping and resilience is reflected by the clusters of “stress management” and “adaptability”. The stakeholders highlighted the necessity of emotional regulation and the ability to perform under pressure as key components of stress management, particularly in elite sports settings. With respect to adaptability, stakeholders stress the importance of shifting strategies and maintaining flexibility during setbacks, allowing athletes to adjust effectively to challenges and unpredictable situations. These coping mechanisms are viewed as essential for long-term success and psychological well-being.

##### 3.2.2.3 Motivation and engagement

The theme of motivation and engagement is covered by two clusters: “intrinsic motivation” and “extrinsic motivation”. The stakeholders named self-determination (representing athletes' internal drive fuels commitment) and task-based motivation (emerging from skill mastery and progression) the two key components of internal motivation. In contrast, extrinsic motivation was described as a construct characterized by coach-driven goal setting and parental encouragement, both of which have been identified as critical factors in sustaining athlete engagement. Moreover, stakeholders noted that external support networks play a crucial role in reinforcing persistence, managing setbacks, and maintaining long-term motivation.

##### 3.2.2.4 Self-management

The theme of self-management is reflected in the clusters of “time management” and “discipline” in the interviews. Regarding time management, stakeholders emphasized the challenge of balancing dual careers, such as academic or professional responsibilities alongside training, while ensuring that structured training schedules support optimal performance. With respect to discipline, the stakeholders described regular practice as a cornerstone of consistency and skill development, while following a structured daily schedule was regarded as crucial for fostering resilience and sustained focus. The stakeholders also highlighted that effective self-management enables athletes to optimize their training while efficiently handling multiple commitments.

##### 3.2.2.5 Social and emotional intelligence

The theme of social and emotional intelligence is manifested in two clusters: “team collaboration” and “empathy”. Regarding team collaboration, stakeholders highlighted the value of peer learning and effective communication in teams, as these foster cooperation and knowledge exchange. In the empathy cluster, the stakeholders commented on the ability to recognize teammates' struggles, which strengthens interpersonal relationships and enhances team cohesion. Moreover, the stakeholders stressed that social and emotional intelligence not only improves group dynamics but also contributes to motivation and performance consistency.

##### 3.2.2.6 Performance preparation

The topic of performance preparation is apparent in the two clusters of “imagery” and “focus and attention”. The stakeholders described the visualization of positive scenarios as contributing to self-confidence. Focus and attention emphasize techniques for blocking distractions and concentrating to ensure that athletes stay mentally focused in high-pressure situations Stakeholders view these psychological strategies as essential to succeeding in challenging situations.

##### 3.2.2.7 Personal growth

The theme of personal growth becomes evident in the two interview clusters of “resilience” and “self-confidence”. With respect to the resilience cluster, stakeholders emphasized the importance of overcoming injury setbacks and achieving emotional recovery from competition failures, reinforcing psychological strength. The self-confidence cluster focused on the role of positive affirmations and the confidence gained through achieving small goals, both of which were seen as integral to maintaining motivation and belief in one's abilities. Many stakeholders highlighted that personal growth is a continuous process shaped by challenges and achievements throughout an athlete's career.

### 3.3 Abductive reasoning on the basis of inductively and deductively derived interview outcomes

A comparison of the inductively and deductively interview analysis outcomes as the foundation for abductive reasoning is summarized in Table 7.

**Table 7:** A comparison of the inductively and deductively interview analysis outcomes.

Aspect	Inductive approach (grounded theory)	Deductive approach (content analysis)
Methodology	Bottom-up, data-driven approach; concepts emerged from expert interviews.	Top-down, hypothesis-driven approach; themes were assigned to the predefined ATDE & PCDE frameworks.
Conceptual focus	Exploratory understanding of mental skills training in youth sports.	Confirmation and categorization of data within established theoretical frameworks.
Main emerging themes	1. Structure & setting 2. Roles & responsibilities 3. Content & domains 4. Processes & interactions	1. Athlete & organizational resources 2. Coach-athlete & peer dynamics 3. Career level transition support & cultural influences 4. Psychological Characteristics & Skills
Boundaries & flexibility	No clear boundaries, overlapping themes, strong interrelationships between concepts.	Clearly, structured within predefined categories of ATDE & PCDE.
Focus of analysis	Identifying the organic evolution of mental skills training through various perspectives.	Examining mental skills training through structural and psychological frameworks.
Key findings	• Importance of organizational anchoring, accessibility, and professional development. • Collaboration and transparency in roles are vital. • Continuous education and interdisciplinary cooperation enhance effectiveness.	• Resource availability (time, personnel, finance) is critical. • Structural programs and policies reinforce athlete development. • Psychological skills (motivation, resilience, time management) are crucial for success.
Abductive reasoning: implications	• Mental skill programs should be embedded strategically in a policy framework • Mental skill programs should be backed-up by a top-down commitment • Mental skill programs need dedicated resources • Mental skill programs need clear roles and responsibilities • Mental skill programs should be accompanied by systematic education of the providers • Mental skill programs need quality regarding content and delivery	

The results of the inductive analysis highlight the importance of practical experience. They reflect the interrelationships between different factors and influencing variables and create a holistic picture of the complexity of long-term mental skill development. In contrast, the findings also highlight the value of deductive content analysis in systematically categorizing participants' experiences into existing frameworks. At the subcategory level, there was almost 88% alignment between the subcategories of the inductive analysis and the ATDE categories. Only 4 out of 33 subcategories could not be categorized. Since mental abilities also formed a separate category in the inductive analysis, they could be compared with the PCDEs at the level of coded segments. Among the 331 segments, 37 (11.2%) could not be classified in the PCDEs. Interestingly, despite the participants' diverse national backgrounds, which spanned North America, Oceania, and various parts of Europe, no clear cultural differences emerged from the analysis. Taken together, the inductively derived themes that are well aligned with ATDEs and PCDEs demonstrate the interconnectedness of structural, cultural and psychological factors in promoting sport and personal development.

Despite the strong alignment of most stakeholder statements and the ATDEs/PCDEs frameworks, however, some statements did not fit neatly into the existing subcategories, for example, process-oriented factors such as education and professionalism. Such unallocated or divergent codes indicate a need for further research and adaptation of existing athlete development frameworks. More specifically, current mental skill development frameworks may be particularly lacking in relation to the themes of “role alignment”, “communication” and “long-term athlete development strategies”, which emerged from our stakeholder interviews. Particularly for these unassigned themes, existing frameworks should be expanded to better capture nuances in talent development and psychological training.

## 4 Discussion

**Figure 5:**
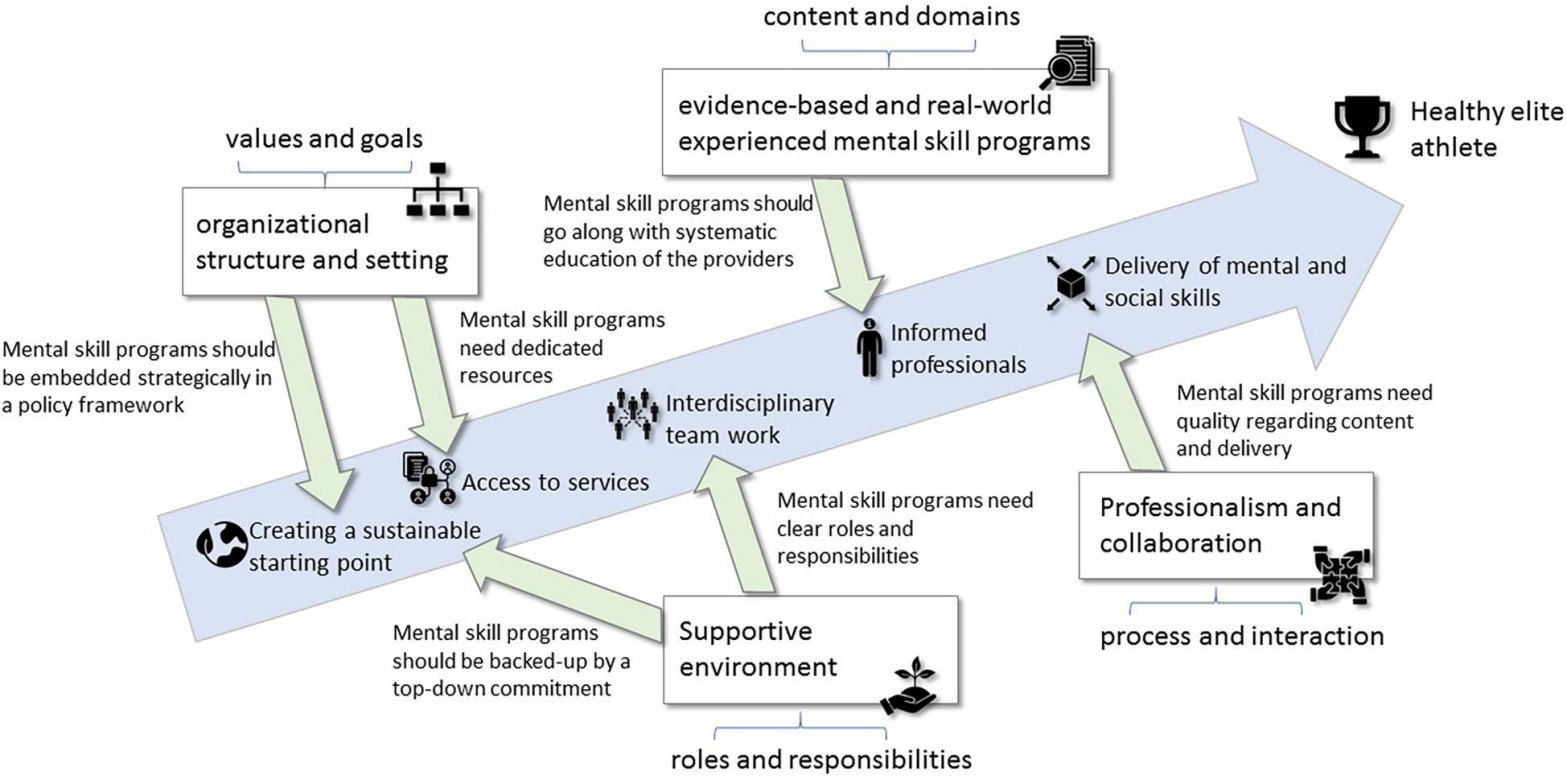
Process model for implementing mental skills programs. The green arrows indicate the abductively derived implications, and the blue arrows indicate key steps for practical implementation.

Figure 5 summarizes the abductively derived implications and key steps for practical implementation per main concept/subconcept that serve as a backbone of the subsequent discussion of our study findings.

### 4.1 Abductively derived practical implications

#### 4.1.1 Mental skill programs should be embedded strategically in a policy framework

To support the long-term nature of a healthy career, mental skills programs should be embedded in an organization's policies. As highlighted by Collins et al. (34), “[…], coaches, researchers, and organizations need to be very clear about what they are working to achieve” (p.342). This clarity needs to be reflected not only in individual goals but also in the overarching strategies of the organization.

A major challenge to achieving this clarity lies in the structural and administrative support within sports organizations. For example, in their study with coaches, Barker and Winter (30) identified the lack of administrative support (athletic director, board of education, and superintendent) as a key barrier. This finding aligns with our results. Both the inductive and deductive analyses highlighted the importance of organizational support for mental skill programs.

#### 4.1.2 Mental skill programs should be backed up by a top-down commitment

Building on this, it becomes evident that a comprehensive and sustainable strategy for mental skills development must be rooted in the organization itself. This requires coordinated effort and commitment from all relevant stakeholders. Indeed, there is often a need for some “active selling” of ideas to promote a more universal buy-in. This is also importantly promoted from the top. The credibility and effectiveness of such programs depend on the support of decision-makers, who provide authority and legitimacy to drive implementation. Without an organizational top-down endorsement, mental skills initiatives often struggle to gain traction.

Stakeholders in our study repeatedly mentioned that decisions about the implementation, development, and sustainability of mental skills programs are heavily influenced by individuals in leadership positions. Given the high staff turnover in sports systems (54–56), any change in personnel often puts the program at risk, mostly in component details but sometimes even in its entirety. It is therefore important that long-term mental development is guided and legitimized by the organization's values and policies rather than resting on the unstable foundations of individual decision-makers.

#### 4.1.3 Mental skill programs need dedicated resources

Both the deductive and inductive approaches identified resource constraints as key barriers to the implementation of mental skill training programs. In addition to time, personal or financial limitations, the issue of accessibility within organizations was also highlighted. These findings align with existing research that identifies time (30, 57, 58), personal (especially access to and availability of sport psychologists) (57, 59), and financial (30, 57, 58, 60) constraints as significant barriers to the adoption of psychological skill training.

As noted by the stakeholders, considerable resources are often invested in the design and development of mental skills programs. While this reflects a strong foundational effort, it also highlights the need for greater consensus on *what* content is essential and *how* it should be delivered to ensure effectiveness. Such coherence is especially important when programs need to impact behavior across a variety of settings, which is almost always the case in any youth sport context. The use of structuring tools such as those in the POP could assist this process by providing greater clarity and alignment of expectations. A more strategic use of time, personnel and financial resources can therefore be an important lever to overcome structural limitations and increase the overall effectiveness of such programs.

#### 4.1.4 Mental skill development programs need clear roles and responsibilities

The inductive findings of our study emphasize the importance of clearly defined roles, responsibilities, and collaborative structures in the delivery of mental skills training. In parallel, the deductive findings highlight key relational and structural factors, particularly the quality of coach-athlete relationships, mentorship, and wider institutional support. Taken together, these findings suggest that fostering structured yet flexible collaboration among all stakeholders is essential for the effective implementation of mental skills programs. Importantly, this need for coherence should be addressed at each age/stage *and* progressively as the athlete develops [cf. horizontal and vertical coherence – Webb et al. (61)].

Support teams often consist of several subdisciplines with different areas of expertise. This requires these people to be able to work together effectively (62, 63). While this diversity brings invaluable perspectives and expertise, it also requires a high level of coordination and understanding. Effective collaboration depends on the ability of these individuals to work together cohesively, despite differing perspectives and roles. This is compounded by the issues of physical resources and performance development in an organization (64).

To address these challenges, clarifying roles and responsibilities at all levels, enabling clear accountability and creating a structured framework are crucial. Assigning responsibilities to team members and knowing who is responsible for what helps anticipate the needs of other team members (65). This helps to optimize processes within the team. Ideally, this will lead to strategic top-down actions, where leadership actively supports and drives initiatives that promote mental health and skills development.

#### 4.1.5 Mental skill programs should be accompanied by systematic education of the providers

The deductive analysis explicitly categorizes mental skills under PCDEs (motivation, resilience, self-management, emotional intelligence, etc.), whereas the inductive approach involves discussing similar concepts within content and domains but with less defined structures. Most of the programs described by the stakeholders focused on specific psychological constructs, such as resilience or a growth mindset (self-control). Furthermore, the inductive results reflect the wide variety of interpretations of Psychological Skill Training (PST) in the literature. In their review, Park and Jeon (66) identified 405 articles on PST including different approaches, strategies and techniques. According to the POP, the development of a broad and adaptable range of psychological skills should be a central aim of mental skills training. The POP advocates a more comprehensive and process-oriented approach that focuses on the explicit teaching, practice and refinement of a range of skills. This structured and focused method ensures that athletes are better prepared to transfer these skills to a wide range of challenges they may face as they develop. Consequently, there is a growing need to embed standardized psychological skills training within the broader framework of organizational development to ensure coherence between individual development and systemic goals. In a resource-constrained environment, it becomes even more important to focus on a core set of skills that are based on both empirical evidence and applied real-world experience.

While performance was identified by stakeholders as one objective, mental health was more frequently emphasized as the primary goal of mental skills programs. This emphasis reflects the growing recognition that mental health not only is essential for the well-being of athletes but also significantly influences their performance and long-term development (67). Within the context of a clearly defined organizational philosophy, mental health emerges as a vital component of mental skill development. Promoting mental health effectively requires thoughtful consideration of cultural factors and contextual influences. Aligning mental health initiatives with the overarching goals of the sports organization can further enhance their impact, contributing to a supportive environment in which individuals are able to thrive (68).

To address both mental health and performance, stakeholders emphasized the need for a clearly defined strategy in the training and teaching of mental skills. A key element is the systematic and ongoing training of coaches, parents, and support staff utilizing a comprehensive set of tools to develop and promote comprehensive skills in athletes (34). Ensuring that all relevant stakeholders are well informed and aligned with the organization's objectives is fundamental for constructing a cohesive support network that underpins the long-term well-being and success of athletes.

#### 4.1.6 Mental skill programs need quality regarding content and delivery

Given their pivotal role in shaping athletes' developmental environments, providing professionals with the knowledge and tools needed to foster healthy and successful mental development is essential. Maintaining and advancing professional standards and scientific evidence remains vital, including understanding the principles of mental skills training, recognizing the signs of mental health challenges, and applying mental interventions effectively. On the basis of findings from our stakeholder interviews and further supporting literature, a lack of knowledge of sport psychology is often the main reason why professionals avoid sport psychology services (30, 57, 69). This highlights the urgent need to also improve the quality of sport psychology education across all levels of sport professionals.

The interviews further revealed that diverse methods and media are currently used to deliver mental skills training. Delivery formats range from group courses to individualized coaching sessions, with the choice of format contingent upon organizational structures, resource availability, and the developmental stage of the athletes (70). Stakeholders consistently emphasized the importance of offering a variety of delivery formats to address different needs and contexts.

### 4.2 Practical implementation steps

An important aspect of applied research is the transfer into practice. According to our study and supporting literature, the following recommendations can be made for practical implementation. The process model (Figure 5) shows the relationship between the main concepts derived from the study and the steps for implementation.

Embedding mental skills training into the strategic and policy frameworks of sports organizations is crucial to achieve long-term impact and sustainability. This is achieved through top-down commitment, structural support, and clearly defined organizational values.

A resource audit should be conducted at the beginning of the planning phase. Anticipating and addressing common barriers related to time management, staff capacity, and budget constraints can improve the feasibility and sustainability of programs, especially in resource-limited settings. Making resources available effectively can help ensure effective access to services and support long-term, sustainable initiatives.

To improve the effectiveness of mental skills training, practitioners and organizations must establish clear roles and responsibilities within interdisciplinary teams and encourage structured yet flexible collaboration. Key strategies include fostering close communication and collaboration within the supporting team and encouraging parental involvement. Additionally, it is important to consider both the athlete's sports and the school environment.

Finally, to support athletes' mental development effectively, sport organizations should prioritize comprehensive education in sport psychology for all professionals involved. This includes systematically training coaches, parents, and support staff not only in the content but also in the effective delivery of mental skills interventions, using a variety of pedagogically sound methods tailored to organizational contexts and athlete needs. Building a knowledgeable and aligned support network is essential for fostering the quality of mental skill programs, and the long-term well-being and success of athletes.

### 4.3 Methodological considerations

This study includes a wide range of perspectives on mental skill development. Using a broad sample of stakeholders from different sports and professions captures many relevant areas of the topic. However, our sample is limited geographically to countries across three continents, which could lead to a Western cultural perspective. Moreover, information on the interviewees' countries of birth (not necessarily their countries of employment) was lacking. This may have further influenced the results due to socio-cultural differences. Finally, using the respondent-driven sampling approach may also have resulted in some selection bias.

This study adopted a constructivist approach, acknowledging that the social and professional backgrounds of the researchers may influence both data collection and interpretation. The research team comprised an international group from Switzerland and the United Kingdom, bringing diverse professional perspectives to the project. The team included one woman and four men. PM is a sport psychologist and PhD candidate; US, a sports physiotherapist, contributed to the project as part of her Master's thesis. DC, RF, and JS are senior researchers with experience in (sport) psychology and sport science.

## 5 Conclusion

Our findings underscore the need for a systematic and sustainable integration of mental skills development within youth sports, which requires robust institutional support, active stakeholder engagement, and interdisciplinary collaboration. To ensure long-term implementation, mental skills development programs must be embedded into the strategic frameworks and policy structures of sports organizations, clarifying roles and responsibilities while enabling the formulation of effective, ideally top-down, actions to support athletes' psychological growth. A central component of this process is the dissemination of evidence-based knowledge regarding healthy and effective mental skills development pathways, alongside corresponding intervention strategies, to all relevant actors within the sports ecosystem. Overall, our findings and the corresponding abductive reasoning reinforce the need for a holistic and integrated approach to athlete development that combines the structural and interpersonal dynamics of talent development environments with a comprehensive psychological skill set. Successful environments should foster athletic, personal, and psychological growth, whereas dysfunctional environments risk an overemphasis on performance at the expense of well-being and long-term development. Nevertheless, such frameworks may need to be developed and refined through practical knowledge and experience.

## Data Availability

The raw data supporting the conclusions of this article are not readily available because their access is restricted in order to protect the anonymity of the participants. Requests to access the datasets should be directed to philippe.mueller@balgrist.ch.
