# Supplementary material for: “Everybody wants to coach it, but fewer know how to do it”: a qualitative study of stakeholders’ perspectives on mental skill development in youth sports
Source: Front Sports Act Living. 2025 Sep 12;7:1633943. doi: 10.3389/fspor.2025.1633943 (PMC12463978; doi:10.3389/fspor.2025.1633943)
Supplement: Supplementary file 1 [file Datasheet1.pdf]

Supplementary Material

Consolidated criteria for reporting qualitative studies (COREQ): 32-item checklist

Developed from:

Tong A, Sainsbury P, Craig J. Consolidated criteria for reporting qualitative research (COREQ): a 32-item checklist for interviews and focus groups. *International Journal for Quality in Health Care*. 2007. Volume 19, Number 6: pp. 349 – 357

**No. Item**	**Guide questions/description**		**Reported on**
**Domain 1: Research team and reflexivity**			
Personal Characteristics			
1. Interviewer/facilitator	Which author/s conducted the interview or focus group?	PM, US	Data collection
2. Credentials	What were the researcher's credentials? E.g. PhD, MD	PM = sport psychologist, PhD-Student; US = sport physiotherapist. master-student;<br>DC, RF, and JS = senior researchers	Methodological considerations
3. Occupation	What was their occupation at the time of the study?	Same as *2. Credentials*	Methodological considerations
4. Gender	Was the researcher male or female?	PM, DC, RF, JS = male; US = female	Methodological considerations
5. Experience and training	What experience or training did the researcher have?	PM has experience in conducting qualitative interviews. US were trained to conduct qualitative interviews (pilot interviews)	Data collection
Relationship with participants			
6. Relationship established	Was a relationship established prior to study commencement?	No relationship	Participants and recruitment
7. Participant knowledge of the interviewer	What did the participants know about the researcher? E.g. personal goals, reasons for doing the research	General outlines of research as described in information letter sent by email. Signed informed consent	Participants and recruitment
8. Interviewer characteristics	What characteristics were reported about the interviewer/facilitator? e.g. Bias, assumptions, reasons and interests in the research topic	No	Methodological considerations

Domain 2: study design			
Theoretical framework			
9. Methodological orientation and Theory	What methodological orientation was stated to underpin the study? e.g. grounded theory, discourse analysis, ethnography, phenomenology, content analysis	Inductive analysis: Grounded Theory/a constructivist paradigm<br>Deductive analysis: Content analysis	Research philosophy and study design
Participant selection			
10. Sampling	How were participants selected? e.g. purposive, convenience, consecutive, snowball	Participants were by respondent-driven sampling	Participants and recruitment
11. Method of approach	How were participants approached? e.g. face-to-face, telephone, mail, email	By email	Participants and recruitment
12. Sample size	How many participants were in the study?	Fifteen	Results
13. Non-participation	How many people refused to participate or dropped out? Reasons?	Nine participants did not answer the invitation for interview and were reminded twice.	Participants and recruitment
Setting			
14. Setting of data collection	Where was the data collected? e.g. home, clinic, workplace	interviews were conducted through Microsoft Teams	Data collection
15. Presence of non-participants	Was anyone else present besides the participants and researchers?	No	Not reported
16. Description of sample	What are the important characteristics of the sample? e.g. demographic data, date	Nation, gender, profession	Results
Data collection			
17. Interview guide	Were questions, prompts, guides provided by the authors? Was it pilot tested?	Interview guide, which was pilot tested twice.	Data collection
18. Repeat interviews	Were repeat interviews carried out? If yes, how many?	No	Not reported
19. Audio/visual recording	Did the research use audio or visual recording to collect the data?	Audio recorded	Data analysis
20. Field notes	Were field notes made during and/or after the interview or focus group?	Yes, during and after.	Not reported
21. Duration	What was the duration of the interviews or focus group?	The average interview length was 49 minutes (range: 34-70 minutes).	Results

22. Data saturation	Was data saturation discussed?	Data, thematic, and theoretical saturation	Data collection
23. Transcripts returned	Were transcripts returned to participants for comment and/or correction?	The transcriptions were not returned to participant	Data analysis
**Domain 3: analysis and findings**			
Data analysis			
24. Number of data coders	How many data coders coded the data?	Two coders	Data analysis
25. Description of the coding tree	Did authors provide a description of the coding tree?	No	Not reported
26. Derivation of themes	Were themes identified in advance or derived from the data?	Derived from the data	Data analysis
27. Software	What software, if applicable, was used to manage the data?	MAXQDA	Data analysis
28. Participant checking	Did participants provide feedback on the findings?	Yes, the results (inductively derived main concepts and categories with brief descriptions of them) were then sent to the participants for reflection	Trustworthiness of the data
Reporting			
29. Quotations presented	Were participant quotations presented to illustrate the themes/findings? Was each quotation identified? e.g. participant number	Quotes presented to illustrate themes and findings Each quotation identified by participant number	Results and Tables 1-4
30. Data and findings consistent	Was there consistency between the data presented and the findings?	Yes	Results
31. Clarity of major themes	Were major themes clearly presented in the findings?	Yes, four main concepts with categories and subcategories	Results and and Figure 3
32. Clarity of minor themes	Is there a description of diverse cases or discussion of minor themes?	Yes, the minor code related to the categories and subcategories are presented	Results and Figure 3
